# EMERGE Modular Robot: A Tool for Fast Deployment of Evolved Robots

**DOI:** 10.3389/frobt.2021.699814

**Published:** 2021-07-05

**Authors:** Rodrigo Moreno, Andres Faiña

**Affiliations:** REAL Lab, IT University of Copenhagen, Copenhagen, Denmark

**Keywords:** evolutionary robotics, morphology evolution, automatic reconfiguration, physical robots, autonomous hardware evolution

## Abstract

This work presents a platform for evolution of morphology in full cycle reconfigurable hardware: The EMERGE (Easy Modular Embodied Robot Generator) modular robot platform. Three parts necessary to implement a full cycle process, i.e., assembling the modules in morphologies, testing the morphologies, disassembling modules and repeating, are described as a previous step to testing a fully autonomous system: the mechanical design of the EMERGE module, extensive tests of the modules by first assembling them manually, and automatic assembly and disassembly tests. EMERGE modules are designed to be easy and fast to build, one module is built in half an hour and is constructed from off-the-shelf and 3D printed parts. Thanks to magnetic connectors, modules are quickly attached and detached to assemble and reconfigure robot morphologies. To test the performance of real EMERGE modules, 30 different morphologies are evolved in simulation, transferred to reality, and tested 10 times. Manual assembly of these morphologies is aided by a visual guiding tool that uses AprilTag markers to check the real modules positions in the morphology against their simulated counterparts and provides a color feedback. Assembly time takes under 5 min for robots with fewer than 10 modules and increases linearly with the number of modules in the morphology. Tests show that real EMERGE morphologies can reproduce the performance of their simulated counterparts, considering the reality gap. Results also show that magnetic connectors allow modules to disconnect in case of being subjected to high external torques that could damage them otherwise. Module tracking combined with their easy assembly and disassembly feature enable EMERGE modules to be also reconfigured using an external robotic manipulator. Experiments demonstrate that it is possible to attach and detach modules from a morphology, as well as release the module from the manipulator using a passive magnetic gripper. This shows that running a completely autonomous, evolution of morphology in full cycle reconfigurable hardware of different topologies for robots is possible and on the verge of being realized. We discuss EMERGE features and the trade-off between reusability and morphological variability among different approaches to physically implement evolved robots.

## 1 Introduction

Evolving the morphology of a robot to better fit a task is a long sought solution. Sims (1994) started this field by evolving morphologies and controllers for virtual creatures simultaneously, but several other works have evolved virtual creatures after his seminal work (Chaumont et al., 2007; Lehman and Stanley, 2011; Lessin et al., 2014; Lipson et al., 2016). Automatically designing robots using artificial evolution techniques helps speed up the robot design process and has the potential of creating novel solutions. In several instances, evolutionary algorithms have demonstrated their ability to find solutions that human designers are not aware of, or that seem counter intuitive (Lehman et al., 2020). The aim of this work is to present a platform designed for performing morphology evolution in hardware that is fast and easy to build and use.

While the optimization of controllers for robots using evolutionary techniques has been extensively studied and demonstrated in real robots (Nolfi et al., 2016), the deployment of evolved morphologies and controllers in reality is still in its infancy. The main difference is that the controllers can be transferred to a robot for testing easily, but this is not possible with morphologies. Once a morphology is chosen, the robot needs to be manufactured. And most works on virtual creatures are infeasible to produce in reality. Nevertheless, having the ability to design and deploy different kinds of robots would be a big advantage for a system that needs to work with minimal human intervention and where the tasks are unknown a priori, as is the case for systems that work in space exploration or sea exploration.

Some works have proposed methods for evolving and transferring robot morphologies to reality easily. Usually most works have left the most basic parts (actuators, passive parts, wiring, sensors, electronic boards, etc.) untouched, some have opted for not drastically modifying the structure of the robot but only specific parts, while others have given more freedom to the evolutionary algorithms for changing the different robot parts altogether (Moreno and Faina, 2020a). Either method is limited by the real morphing or fabrication method available. Some of them offer more freedom to the evolution, that is, robots have a higher range of shapes for their morphologies. While others limit the available shapes for the evolution. Shape limitation makes the process more computationally tractable as there are fewer variables to modify at any given design run, which also makes finding good results quicker.

One widely used method for building evolved morphologies is 3D printing. This technology is used for systems that can achieve more morphological variability. It is a flexible fabrication technology that can create a great deal of different parts in different shapes and sizes from the same basic materials and in an automatic fashion. These parts are later joined together with other components to create the final robot to be tested. Each part can be tailored for each morphology and dimensions and tolerances depend on the specific process, some of them can even reach sub-millimeter precision. The first ones that demonstrated that the evolved robots can be manufactured were Pollack and Lipson (2000). They used 3D printing to manufacture the mechanical parts and manually assembled the motors and wires. Similarly, Samuelsen and Glette (2015); Auerbach et al. (2014); Vujovic et al. (2017); Buchanan et al. (2020) also used 3D printing for manufacturing evolved robots.

Surprisingly though, many works that use 3D printing limit the kind of shapes that can be created or modified inside the robots. The main reason for this is that these parts must permit their assembly with the rest of components of the robot, and thus must comply also with specific sizes and weights that can be moved with, for example, electric motors. The capacity of the 3D printers to accommodate the size of the parts can also limit the size of the robots. Having parts tailored closely to specific morphologies in turn, creates a situation in which parts cannot be reused for other robots in the test, leading to waste of material and reprocessing. The assembly process is also a time consuming task, in some cases in the order of days, that must be repeated for every new morphology, moreover, printing can take hours depending on the part complexity.

Some recent works have opted to only change the length of very specific parts inside a robot (Nygaard et al., 2020a). This, of course, limits greatly the morphological space that can be reached by the robots in reality, but makes changing shape a very quick process. The mechanism used must be able to lock in place, so the movements of the robot do not affect the morphology being tested at the moment. Placement of shape changing mechanisms inside a robot opens the door to online shape changing and evolution, much in the same direction as origami robots and soft-robots that use shape change as a mean to achieve a task (Shah et al., 2020). However, adding these kind of mechanisms adds complexity to the robot. Shape changing mechanisms must be driven by the internal battery and control systems of the robot, which also implies more wiring. Additionally, the placement of the mechanisms in the robot can make the whole structure more susceptible to contact with the environment and create more points of failure, rendering the robot more fragile overall.

Modular robots encapsulate functionality (sensors, actuators, processors, energy, etc.) in compact units called modules (Stoy et al., 2010), which connect to each other through specially designed reusable connectors and that can be rearranged in different ways to create different morphologies. As the modules are already built, modular robots speed up the assembly of new robot configurations with respect to other approaches, down to using only minutes. Wiring, for example, is virtually eliminated when reconfiguring these kinds of robots, as connectors are able to route power and control signals. Multiple modular robot designs have been developed over the last decades (Yim et al., 2007a; Faiña et al., 2015; Ahmadzadeh et al., 2016; Zappetti et al., 2017; Yao et al., 2019), but only some of them have been used to evolve morphologies in simulation (Marbach and Ijspeert, 2005; Chocron, 2007; Veenstra et al., 2017). And very few have actually built physical robots (Faíña et al., 2013; Auerbach et al., 2014; Brodbeck et al., 2015; Liu et al., 2017; Hale et al., 2020). In addition, some of these works combine 3D printing with modularity to obtain a bigger morphological space and improve the reusability of some components (Auerbach et al., 2014; Hale et al., 2020).

A special type of modular robots, self-reconfiguring modular robots, can even reconfigure without external intervention automatically (Yim et al., 2007a; Stoy et al., 2010). However, the complex mechanisms used for this purpose usually slow the process down. Moreover, they imply the use of specialized parts, e.g. mechanical latches (Davey et al., 2012), which add wiring and weight to the module and that can fail in the docking or undocking process, as in the case of shape changing robots. The self-reconfiguration process (the sequence of actions for the self reconfigurable modules to go from one morphology to another) is algorithmically difficult to generate while maintaining all modules connected to the same body and it could not exist for certain cases (Sucan et al., 2008; Stoy et al., 2010) or suboptimal solutions are found. Mobile self-reconfigurable robots are able to partly overcome this limitation by disconnecting their modules from the same body and moving them through locomotion to their next designated space. However, depending on the robot self-reconfiguration mechanisms, external helper tools (e.g loads) or modules are needed to complete a reconfiguration action (Liu et al., 2019).

Modular robots present interesting properties for implementing evolved robots. Modules can be replaced when damaged or repaired separately. Building several identical modules helps in reducing costs and assembly time. Nevertheless, connectors impose tougher restrictions on module positions in the structure, reducing the morphological space. In addition, connectors occupy space making the robots bigger and heavier than in other approaches, and this can limit some tasks. Yet, as mentioned, a reduced search space can also speed up the finding of well-performing robots, a desirable feature for real systems. Additionally, the mechanical and electrical connectors used in the modules can impact the assembly process and make it less difficult.

Even if robots are feasible to build in reality, it is necessary to decide where the evolution will be performed. In short, there are two main approaches to achieve controllers or morphologies for physical robots. The first one evolves the controllers or designs using simulation tools and then transfers the best ones for testing in the real platforms (Hiller and Lipson, 2011; Koos et al., 2012). The second one avoids simulations and performs the evolution directly in hardware (Floreano and Mondada, 1998; Shen et al., 2012; Faíña et al., 2017; Heijnen et al., 2017). The first approach is the most common as evolutionary techniques are population-based tools which need hundreds if not thousands of tests to work. Thus, running these tests in reality is a very demanding task that requires a lot of human hours and effort. Furthermore, badly configured controllers can damage joints and the repeated motion through tests can end up breaking up the actuators. However, evolving in simulation and then transferring to reality has an important drawback, the reality gap. This happens because the evolutionary algorithm can exploit approximations and badly modeled phenomena of the simulator to achieve high fitness. Therefore, the simulation performance of the robots does not correspond to its performance when implemented in the physical world. Of course, evolving not only the controller but also the whole robot morphology adds new levels of difficulty.

## 2 Evolution of Morphology in Full Cycle Reconfigurable Hardware

One road that opens with the use of modular robots for morphology evolution and transfer systems is the possibility of using external robotic manipulators to automatically rearrange the robot modules. This has the advantage of freeing human resources and of running most of the evolutionary process, if not all of it, in reality, as most of the hard work will be done by a machine. Different works have demonstrated the feasibility of different parts of this specific approach. For example, Brodbeck et al. (2015) use pre-made modules joined together by a robot manipulator with hot glue. Parts can be arranged and connected in more arbitrary ways than in other approaches, but somebody must manually separate them and eliminate the glue for parts to be reused in building other robots. Hale et al. (2019), Hale et al. (2020) mix the use of a robotic manipulator handling standard pre-made modules containing actuators and sensors, which they call *organs*, with custom 3D printed parts designed by artificial evolution. Robots can also be assembled automatically and even wired together automatically, as each organ contains standard electrical connections. However, at the same time, organs are latched into the 3D printing chassis, leaving small room for doing automatic disassembly. Even if the pre-made organs accelerate the assembly of different robots, the 3D printing process to obtain the frame slows everything down and makes at least part of the robot not reusable.

To the best of our knowledge, the full cycle evolution without human intervention, i.e. an evolutionary process in hardware that is capable of configuring, testing, and reconfiguring robots to start over again, has only been implemented for morphological variations in the length of the limbs of a robot (Nygaard et al., 2020b). Until now, it has not been achieved for other 3D morphologies that can more drastically reconfigure the arrangement of the robot parts, that is, that have more topological variability.

**FIGURE 1:**
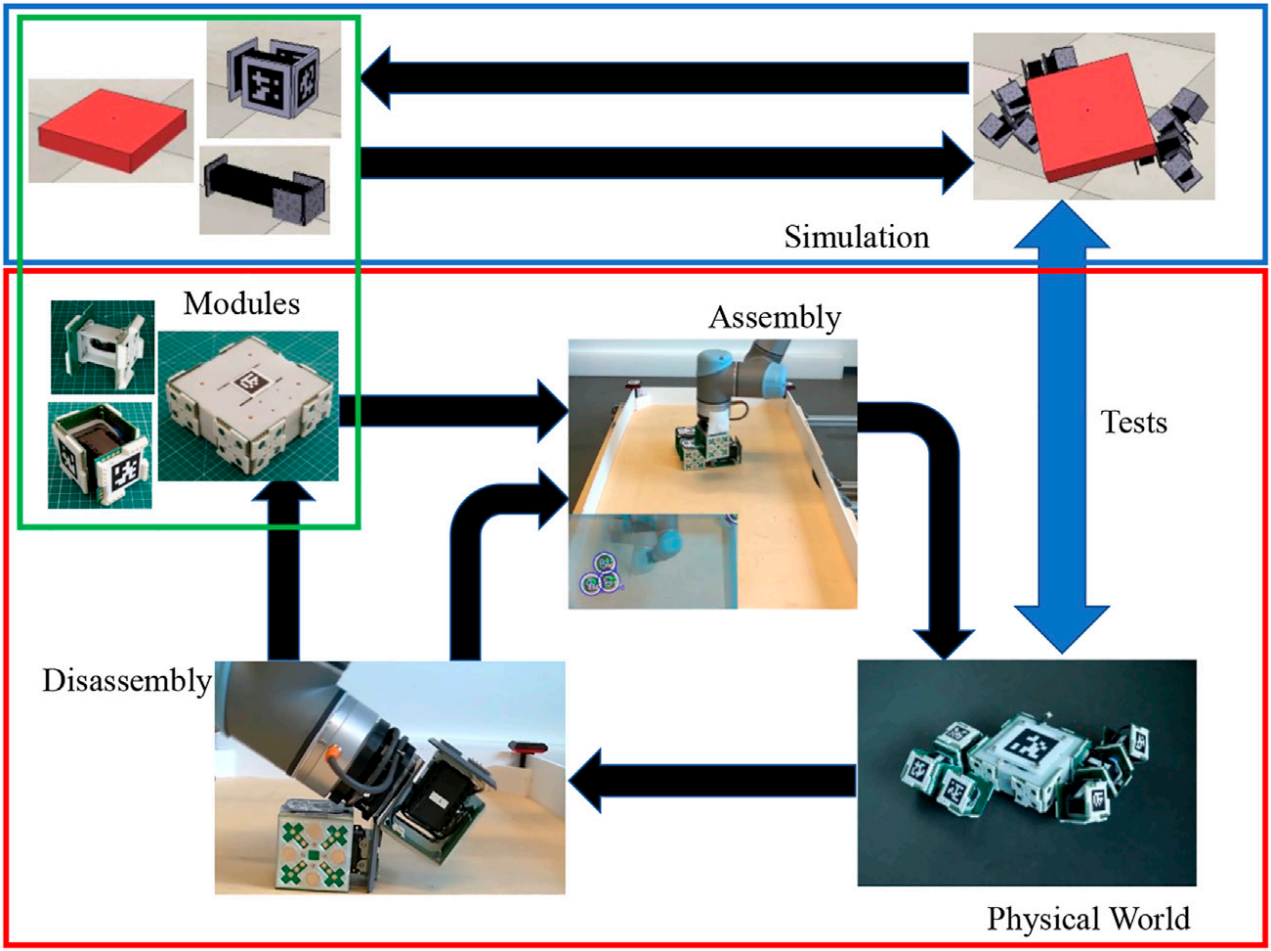
Automatic evolution of morphology in full cycle reconfigurable hardware, with assembly disassembly process, for the EMERGE system using a manipulator with a passive gripper. A set of EMERGE modules is assembled using a robot manipulator with a passive magnetic gripper. The assembled morphology is tested and then disassembled by the same manipulator. The disassembled modules can be stored or reassembled in a new morphology. The system can run an evolution in hardware or use a mixed approach by using results from a simulation.

To achieve an evolution of morphology in full cycle reconfigurable hardware, this work presents a platform for morphology evolution and transfer to reality: EMERGE (Easy Modular Embodied Robot Generator). EMERGE is based on modular robots and it eases the reconfiguration process enough so that an external manipulator could run a full cycle process of reconfiguration and testing, i.e. assemble modules in morphologies, test the morphologies, disassemble modules and start all over again. Our approach using the EMERGE system is summarized in Figure 1.

This work describes all the necessary parts to implement this approach: mechanical design of the EMERGE module, extensive tests of the modules by first assembling them manually, and automatic assembly and disassembly tests.

The EMERGE module has the following advantages: 1) easy and fast to build, 2) fast to reconfigure manually or by an external manipulator, and 3) resilient against self-collision (Section 3). This article describes the latest version of the EMERGE module, which is based on our preliminary work (Moreno et al., 2017). Specifically, the new contributions are 1) an improved design that allows placing markers on the connection faces to localize the modules (Section 4), 2) validation of our claims about EMERGE being easy and fast to build (Section 5) and 3) a new tool that helps to manually assemble morphologies using augmented reality (Section 4.3).

Using EMERGE modules, we evolve morphologies in simulation and test them in hardware. While five evolved robots have been implemented in (Liu et al., 2017), the test presented here 1) documents the assembly time for all the morphologies and shows that the platform can speed up the robot reconfiguration even in the case of being used by an human operator, 2) validates that the modules can resist extensive evaluations in hardware and are resilient against self-collision, 3) reports the signs of wear in the modules and 4) analyzes the reality gap of the EMERGE system methodically.

For the sake of completeness, in Section 6, we summarize the previous assembly and disassembly tests with an industrial manipulator, which were performed in (Moreno et al., 2018). These tests were carried out with the old design, sometimes without tracking markers, and presented severe limitations, which are fixed with the new design. However, they show that autonomous evolution of different topologies for robots in hardware is just a step from being realized.

## 3 EMERGE Design Principles

The EMERGE modular robot has been designed with the following goals:

1) Easy and fast to manufacture: We need several modules to build a robot, so the time spent in manufacturing modules should be minimized and complex operations should be avoided.
2) Easy and fast to reconfigure: The evolved morphologies should be assembled and disassembled without special tools and in seconds.
3) Resilient against self-damage: Evolved morphologies and controllers could have behaviors were their modules collide against each other. The robot should be able to handle these situations without damaging its own modules.
4) Easy to maintain: Worn out parts or broken parts need to be easily replaceable.

One critical design decision in the EMERGE system, that also helps in simplifying the module design, is to skip complex self-reconfiguration mechanisms completely. Instead, we have chosen to base the automated reconfiguration on an external manipulator. This has three main advantages. The first two have been mentioned in the introduction: First, it simplifies the connection mechanism of the modules and avoids intricate designs as self-reconfigurable robots add specialized parts that must be driven independently in the module and that add failure points (Davey et al., 2012); Second, the sequence of actions for self reconfiguration is difficult to generate (Sucan et al., 2008; Stoy et al., 2010) and often sub-optimal solutions are found. Mobile robots are able to overcome some self reconfiguration limitations with the use of external tools (Liu et al., 2019). By having an external manipulator, this process is trivial as, in the worst case scenario, the manipulator could disassemble the robot completely, without having to maintain a main body, and assemble the new one. The complete module-manipulator system as a whole can be considered a different type of self-reconfiguration system. And finally, the external manipulator approach also allows us to just have one degree of freedom (DOF) per each module, which reduces the number of components needed.

EMERGE design uses simple passive magnetic connectors that are used to assemble, test, and disassemble different robot morphologies quickly by human hands. The auto-aligning feature of the magnetic connectors can also be exploited by simpler external agents (e.g., robotic manipulators) to automate the reconfiguration process. The external agents will need to identify the modules easily to reconfigure them. Thus, we have included a fiducial marker at each connector. They can be used by the automatic reconfiguration system but they can also be used to double check that the robot has been built correctly during manual assembly.

The magnetic connectors provide another important advantage when testing evolved robots: if the forces applied through the connectors are excessive, the magnetic connectors disconnect and the modules do not break. Thus, the modules are resilient to collisions between different limbs of the robot. Additionally, EMERGE shares power and communications through its connectors. This avoids cables between modules that could be tangled in some morphologies and controllers.

The EMERGE system aims to make module building as simple as possible by using commercially available components that can be assembled to each other with 3D printed parts and printed circuit boards (PCBs). Some modular robot prototypes use specially designed motors and drive systems (Davey et al., 2012). In addition, even when sketches or drafts of the modules are available, custom designs make modules difficult to build, as specialized steps may require special training. EMERGE modules are designed around a single servomotor that can be daisy-chained to similar motors, again simplifying wiring. Furthermore, we have also opted to have an electronics free version, where all the control is performed by the off-the-shelf components.

Making a modular robot module as easy as possible to build, with no complicated mechanisms or wiring, is a must for speeding up deployment. Easy to produce modules can be assembled quickly for different types of applications or can be stored to be shipped with other robotic systems in different missions. Additionally, using off-the-shelf components not only lowers the bar for non roboticists to use the robot in their projects or research but also reduces maintenance time as parts can be swapped in damaged modules. Moreover, simplifying the building process enables large quantities of modules to be produced, making it possible to test systems with several modules.

## 4 EMERGE Modular Robot

### 4.1 EMERGE Design

**FIGURE 2:**
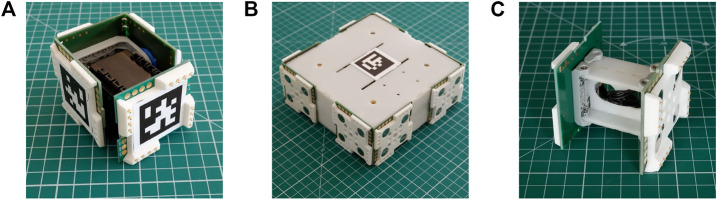
EMERGE modules **(A)** The active EMERGE module **(B)** the base module and **(C)** the link module. The modules can be connected together to build robots with different morphologies. Their magnetic connections allow a quick assembly of the modules, which is useful to test evolved morphologies and controllers. Fiducial markers make them easy to track by an automatic system.

The EMERGE modular robot is an open-source robotic platform^1^ designed to be easy to build, maintain and modify by anyone, that allows fast assembling of evolved robots (Moreno et al., 2017). In the current design, the system comprises an actuated module, a base module and a link module (see Figure 2). However, modules with different designs, even with more complex heterogeneous features like sensor modules, could be created in the future and added to the platform.

The actuated EMERGE module has only one rotational degree of freedom, which is controlled by a servomotor, and four connection faces. The base and link modules are passive (they do not have any DOF) and they have eight and two connection faces respectively. Different robots can be built quickly by connecting modules through their connection faces.

The magnetic connectors are in charge of maintaining the mechanical connection between modules. They are made of a 3D printed part that holds four neodymium magnets (ø12 × 3 mm, N45) and a PCB with electrical contacts. The connectors are low profile (5.2 mm in height). In order to reduce the number of magnets needed and the cost, we decided to have gender connectors^2^. All the magnets in one connector have the same polarity and the polarities of the male and female connectors are opposite. Therefore, male connectors are attracted to female connectors. In addition, the 3D printed part of the male connector has protrusions that mate holes in the female connectors. When two modules are connected, the protrusions and holes are interlocked and they avoid the disconnection from shear forces. The pullout force of the connector is 58N, which is enough to keep the modules connected. In most practical applications, disconnections are caused by the torque applied to the connectors. The connectors can hold up to 1.2 Nm in static applications, but they can be disconnected with lower torque under vibrations. As discussed in Section 3, magnetic connectors provide a quick and practical way of assembling evolved robot morphologies. They also protect the modules from damage when forces and torques are high enough to disconnect parts of the robot, making the system fail in a predictable and robust way and only requiring the modules to be reconnected to run again. Using magnets also allows tags to be placed on top of them without affecting the force of the connection too much.

The PCBs route electrical power and communication signals. The male connectors have spring pins to transmit power and communications from one module to another. These spring pins are in contact with gold plated pads on the female PCBs when the modules are connected. Spring pins ensure that the faces keep electrically connected to each other in the case of small misalignments or vibrations. There are four signals that are shared: power (ground and +12 V), a bus data line to communicate with the motors and an extra connection that is not used at the moment. These four lines are shared in each of the four protrusions of the male connector, which makes the system robust against partial disconnections (one edge of the connector can keep everything connected when the module is being disconnected by external torques).

As the EMERGE system has been designed to be reconfigured by an external manipulator, the connectors need to be easily identifiable. EMERGE modules have been designed to leave the center of their connector free of obstacles in order to place fiducial tags. This is an improvement over previous versions (Moreno et al., 2017) in which fiducial marks interfered with one or more connectors. AprilTags have been selected as they are robust and several tags ids are available (Olson, 2011; Wang and Olson, 2016). Specifically, we use the 36h11 family, which provides 587 different tags. Thus, we can have a unique tag for each connector. The tags are placed in ascending order in each module face starting from the male connector. All tags *Y* axis point in the same direction to maintain consistency and make identifying the orientation of the module easier. Tags can be printed on a sheet of 40 × 40 mm labels and simply glued onto the center of the 3D printed face. This also makes the tags very easy to replace in case of damage.

Tag identification starts with id 0 for the base module and four consecutive tags are assigned to each subsequent module. The tags assigned to a module are organized in ascending order and positioned clockwise (viewed from the top of Figure 2A) in each connecting face, starting with the male connector.

**FIGURE 3:**
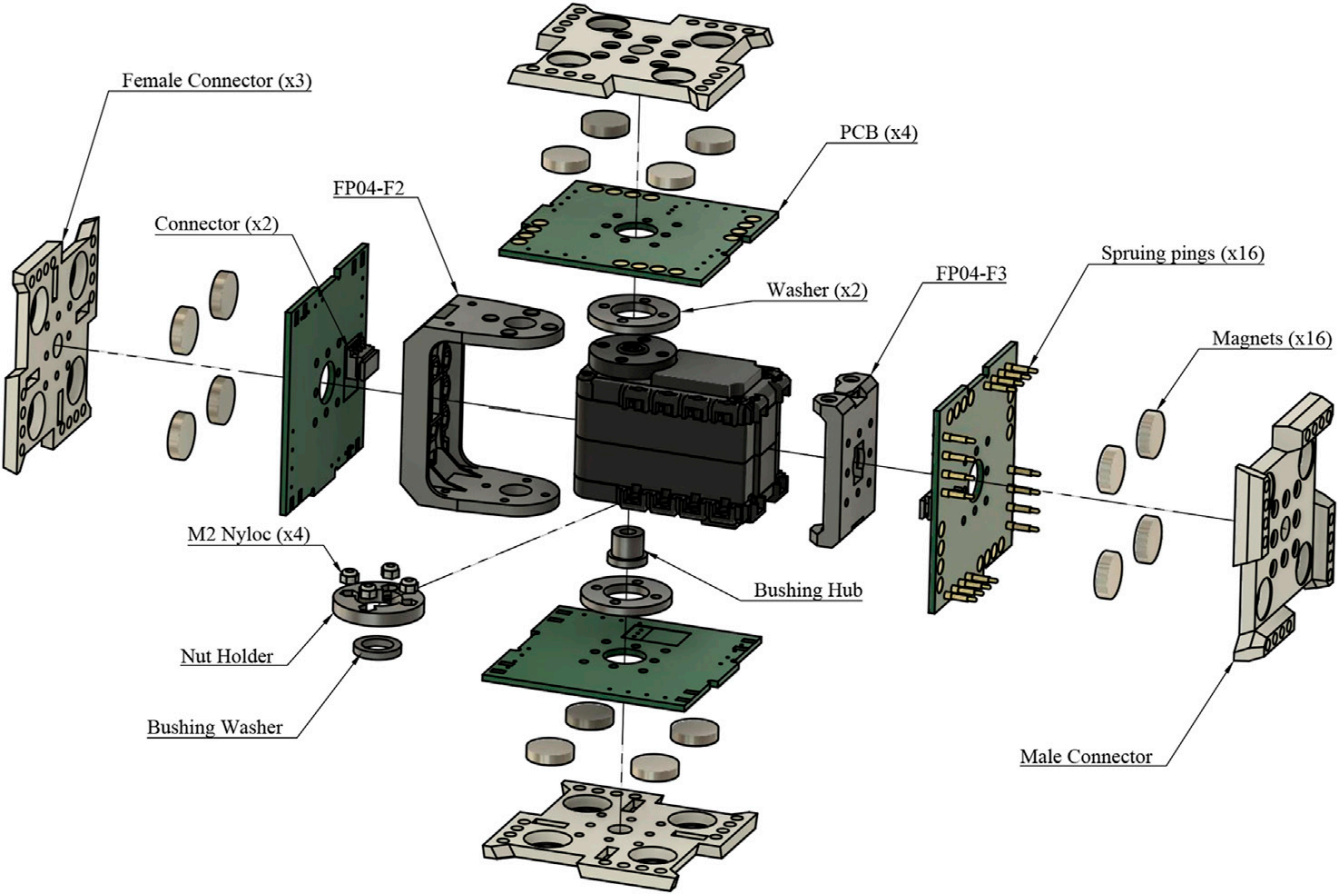
An exploded view of the EMERGE Module. The screws or rivets used to join the four connectors and the brackets (FP04-F2 and FP04-F3) have not been included for the sake of clarity.

The active EMERGE module, which resembles a small cube (See Figure 2A), has a servo motor attached to a pair of off-the-shelf brackets. The male connector is attached to one of the brackets and fixed to the motor. Three female connectors are attached to the other bracket forming an “U” shape and all of them are attached to the rotating part of the servo motor. The PCBs of the female connectors have finger joints interlocking to increase their rigidity. In addition, these PCBs have pads soldered together also to increase the rigidity and share electrical signals and power without connectors or cables. An exploded view of the module and its minimal components is shown in Figure 3. The 3D printed faces and PCBs are secured to the motor brackets through M2 screws and nyloc nuts. Optionally, the male connector and the opposite female connector can be secured by 2 mm rivets to speed up the assembly and avoid loosening. The U shaped subassembly (the three female connectors and the bracket) and the bracket where the male connector is attached can be removed from the motor as they are fastened using M2 screws and nuts. This allows access to the motor gearbox in case of damage.

Two servo motors can be chosen for the module: an AX-12 A or an AX-18 A motor. The main differences are the cost and speed of the motor. We have used AX-18 motors as we had them available. These motors can be daisy chained and have two electrical connectors. Two off-the-shelf cables are used to connect the male and one of the three female connectors with the motor. Thus, electrical connection between all the connectors and the motor is ensured. And therefore, a central controller can be connected to any connection face in order to communicate with any module attached to the robot.

In this paper, we are presenting the simplest version of the EMERGE module, which is the most accessible one and the fastest to build. However, we have developed another version where the PCBs of the connectors house a microcontroller and that makes modules able to control themselves and communicate with other modules via CAN bus (Hernández et al., 2018).

The aim of the base module is to provide a place where we can hold a battery and a centralized controller for the motors. Therefore, the system would not need any cables as the power is distributed through the connectors to the modules. This module is built with eight female connectors grouped in pairs at four corners. Each corner has a 3D printed bracket that provides rigidity and the corners are joined by 2 mm laser cut plates made of Polyoxymethylene (POM). The plates are joined to the connectors with M2 screws and nyloc nuts. Finally, a top and bottom POM plates with finger joints are attached to the 3D printed brackets with M5 screws.

Finally, the link module can be used to extend the distance between the modules. It contains a female connector on one side and a male connector on the other side. Each connector is attached to an off-the-shelf bracket and the two brackets are joined to three 2 mm POM plates with finger joints and screws. The length of the module can be adjusted by cutting new POM plates.

The main features of the three kinds of modules can be seen in Table 1.

**TABLE 1:** Main features of the EMERGE module.

Feature	Emerge version		Base	Link	Unit
	AX-12A	AX-18A			
Overall dimensions	78 × 63 × 55		149 × 149 × 55	55 × 55 x L^a^	mm
Module weight	195	196	502^b^	79^a^	g
Motor torque (12 V)	1.5	1,8	—	—	Nm
Motor speed (no load, 12 V)	59	97	—	—	rpm
Range of motion	±100		—	—	degrees
Resolution	0.29		—	—	degrees
Connector torque	1.2				Nm
Connector axial force	58^c^				N
Modules cantilevered under gravity	3		-	7^a^	Modules
Cost	65	100	46.5^b^	9.5^a^	€

a Links can be built in different lengths, the data shown here is for a length of 57 mm.

b Without battery and controller.

c Not measured. It has been obtained theoretically with the data of the magnets and their air gap from https://www.supermagnete.dk/adhesive-force-calculation/.

### 4.2 Module Building Process

Most of the parts are off-the-shelf and the rest can be easily externalized or manufactured in a fablab or workshop. The bill of materials of the active EMERGE module is shown in Table 2. The 3D printed parts can be manufactured using Fused Deposition Modeling (FDM) or Selective Laser Sintering (SLS). In our case, the connectors were manufactured in SLS by an online 3D printing service. The 3D printed washers and nut holders were 3D printed in polylactic acid (PLA) using a FDM printer at our workshop. The PCBs were ordered to an online electronic supplier. When all the parts are in house, the modules can be built without advanced tools: a soldering iron and a few screwdrivers are enough. To speed up the assembly and avoid errors, we have designed jigs that allow us to assemble the magnets with the right polarity in the face connectors and hold them together while it is assembled to the motor. An additional jig is employed to keep the spring pins straight while soldering them to the PCB. Rivets can be used in two of the faces to speed up the process and avoid the loosening of the faces, but screws and nyloc nuts can also be employed. The basic steps to assemble the module and the time required for each step are as following:

1) Solder the spring ping and electric connectors to the PCBs (6.5 min)
2) Attach the bracket to the motor (3.5 min).
3) Assemble the male connector and attach it to the bracket (4 min).
4) Assemble the three female connectors (2 min).
5) Attach one female connector to the hinge bracket (1.5 min).
6) Connect the cables to the motor and male connector (0.75 min).
7) Attach the second female connector to the motor hinge (5.5 min).
8) Attach the third female connector to the motor shaft (3 min).
9) Solder the PCBs together (2 min).
10) Connect the cable to the female connector (1 min).

**TABLE 2:** Bill of Materials of an EMERGE module.

Part	Quantity	Method	Price unit/line (€)
Male connector	1	3D printed	2.8^a^
Female connector	3	3D printed	1.7/5.2^a^
Whasher	2	3D printed	0.03^b^
Nut holder	1	3D printed	0.03^b^
PCBs	4	PCB order	1.4/5.7
Magnets ø12 × 3 mm	16	Off-the-shelf	0.4/7.0
Motor (AX-12A or AX-18A)	1	Off-the-shelf	40 or 70
Spring pins	16	Off-the-shelf	0.4/6.7
FP04-F2 bracket	1	Off-the-shelf	0.65
FP04-F2 bracket	1	Off-the-shelf	0.65
Bushing hub and washer	1	Off-the-shelf	0.73
Cables (60 and 140 mm)	2	Off-the-shelf	1.3/2.6
Screw and nuts	Several	Off-the-shelf	1.6
Stickers	4	Printed	0.01/0.04
Total			65€ (AX-12A) or 100€ (AX-18A)

a These parts were manufactured using SLS by a 3D printing service. Their cost could be reduced by printing them in a FDM 3D printer.

b These parts were manufactured using FDM in our workshop. They can be printed in less than 5 min and their cost were calculated based on the amount of PLA filament needed to 3D print them.

Thus, a module can be built in half an hour.^3^

### 4.3 Manual Reconfiguration Process

With the purpose of testing the EMERGE design in reality, we repeatedly assemble and disassemble robot morphologies by hand and test them several times. Manually assembling a robot built with homogeneous modules is error prone and time consuming. First, all the modules look the same and one needs to carefully check in which face the next module should be connected. Furthermore, it is easy to assemble a module flipped 180° around its axis, which results in the module actuation being in counterphase with the real one. Finally, the right controller signal needs to be sent to the motors of the robot. Each motor has its own id in the communication bus and thus, it is necessary to know the specific id for each module of the robot. The id of each module could be enforced by the assembly process, but this causes issues if that specific module is damaged or not available. We have chosen to assemble the robot with any module available and then associate that id with the controller of the module in that position. In order to reduce errors, we have developed a tool to assist with the manual assembly process.

**FIGURE 4:**
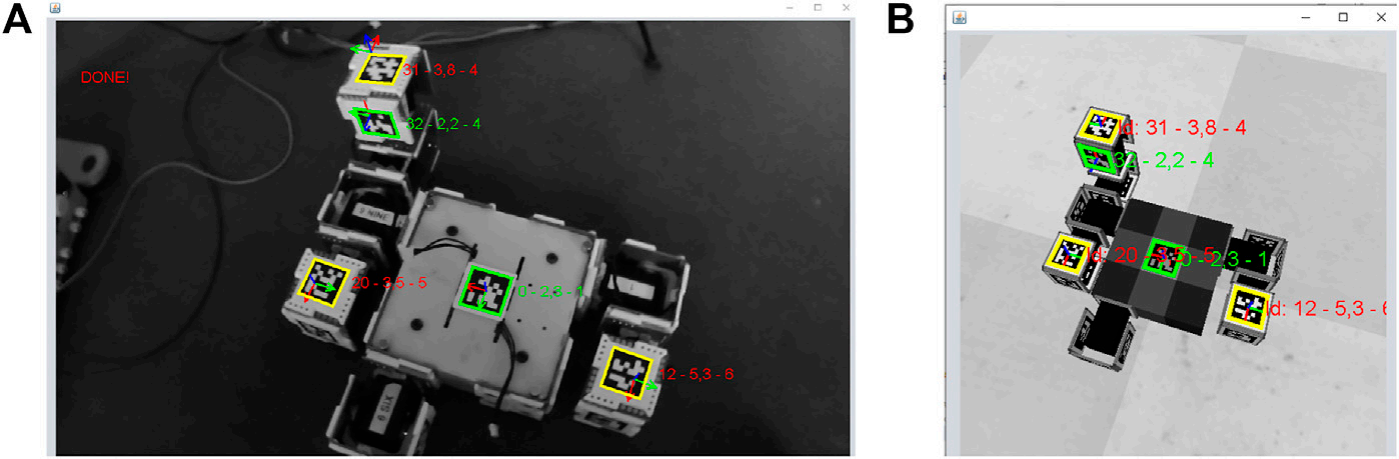
The assembly tool when building a robot with EMERGE modules **(A)** The image from a handheld camera controlled by a user **(B)** The image from the simulation taken by a virtual camera with the same position and orientation as the handheld camera. All the recognized Apriltags are highlighted indicating if they are placed correctly (Green means “OK”, yellow “double check” and red “not placed correctly”) Additionally, the id of the tag, and the distance and orientation errors are shown.

The tool uses augmented reality to inform the researcher where the next module to assemble should be placed and if it has been placed and oriented correctly. The user starts the process by introducing the genotype of a robot (morphology and controller) and the tool assembles the first module in the simulator. Then, the user places the first module on the arena and points at it with a handheld camera. Automatically, the tool finds the Apriltag of the first module and extracts its position and orientation. This information is used to automatically move the camera sensor in the simulator to the same position and orientation as the handheld camera. Thus, the researcher can inspect the robot being assembled from his or her point of view. Then, the researcher will place the second module in the field of view of the camera and the tool will identify the id of the module based on the Apriltags on the faces of the module. The second module is added to the simulator in the right position and the recognized Apriltags of the module are shown in the webcam and simulator views. When the position and orientations of the module are within a threshold (because the module has been assembled correctly), this information is shown and the color changes from red to green to inform the user (See Figure 4). This process is repeated until the assembly ends.

At the end of the assembly, the list of identified modules and their ids are returned by the program. This list of ids can be used by a robot controller to generate the same movements as in the simulation.

## 5 Testing Evolved Morphologies

As a previous step to automatically reconfiguring robots, we validate the different features of the EMERGE module design by evolving morphologies and controllers for robots in simulation and transferring and testing them in reality manually. A modular robot evolutionary framework, the EDHMOR system (Faíña et al., 2013), is in charge of evolving the robots in simulation. We performed 30 independent runs, and the best robot found in each run is manually assembled and tested in reality 5 times. Then, each robot performance is compared to the performance of its simulated counterpart.

### 5.1 Experimental Setup

The EMERGE robot morphologies are evolved with the EDHMOR framework (Faíña et al., 2013). Robots are codified using a direct encoding where the morphology is represented as a tree. Similar to other works (Cheney et al., 2018), the framework has a simple mechanism to force and protect innovations: It forces morphological innovations by adding modules to the robots in a growing phase, which is followed by 2 phases where the morphology can be adapted to the new modules and one adaptation phase for the controller. After them, two pruning phases discard the modules of a robot that do not contribute to the fitness. Finally, a replacement phase replaces the worst individuals in the population with random individuals and symmetric robots of the best individuals (randomly selecting a limb attached to the base and making its reflection through the XZ or YZ planes). If it is not possible to generate a symmetric robot, a random individual is created instead. All these phases are repeated cyclically until the evolution ends.

In this experiment, the base module is always used as the starting module. EMERGE modules (AX-18 A version) are attached according to the genotype. Link modules are not used. The robots are controlled by sinusoidal controllers where the phase is the only free parameter, adjusted by the evolutionary algorithm. The output of the controller is applied directly as a position target for the module rotational actuator. The amplitude of the sinusoidal signal is set to 90° and the angular frequency to 1. Therefore, every 2π s, a full oscillation is performed.

In order for EDHMOR to test robot morphologies in simulation, the CoppeliaSim simulator^4^ is used. The EDHMOR system calls the simulator API to build the robots from simulated modules and joins them together using simulated force sensors. These are special types of joints that can break when the forces and torques applied to them exceed a preset limit for a consecutive number of times. In our setup, the force sensors break when the force exceeds 1 Nm more than seven consecutive times. The torque is less than the real one (1.2 Nm) to take vibrations into account.

Robots can jitter before they converge to a stable gait. There are two main reasons for this behavior. First, the robots start with all the modules at 0° and their initial target positions can be far away, which causes aggressive movements of the modules at startup. Second, the robots start with at least one of the modules in contact with the floor, but in that position the robot is not statically stable. To discard these effects, the system allows the robot to move for a total of 6 s before it starts to measure its fitness. The robots are simulated for 38 s. The planar distance traveled by the center of the base module is calculated from the initial time for fitness evaluation (second 6) to the final time (38 s). The fitness of each robot is calculated as this distance multiplied by a penalty for every module that is disconnected through the test. The penalty is designed to discourage module disconnections and the overall fitness aims to obtain robots that travel as far as possible without breaking apart. Equation 1 shows the fitness as calculated by the evolutionary algorithm.

$$F=\sqrt{{\left(x_{\text{final}}-x_{\text{initial}}\right)}^{2}+{\left(y_{\text{final}}-y_{\text{initial}}\right)}^{2}}\times {\text{penalty}}^{\#\text{ broken connections}}$$ (1)

Each evolutionary run in the simulation has an initial population of 40 individuals and runs until 25,000 evaluations have been performed. A total of 30 repetitions are done. Table 3 shows other parameters relevant to the evolution in simulation. Evolution and simulations are run using the High Performance Computing (HPC) cluster at the IT University of Copenhagen (ITU).

**TABLE 3:** Simulation experiments parameters.

Parameter	Value
Evaluations	25,000
Population	40
Repetitions	30
Variants tested in growing phase^a^	3
Variants tested in morphological phase^a^	3
Variants tested in control phase^a^	10
Symmetric robots generated in replacement phase^a^	5
Random robots generated in replacement phase^a^	5
Controller mutation probability	0.4
Initial time for evaluation (s)	6
Max allowed time (s)	38
Broken connection penalty	0.8
Actuator range (radians)	$\left[-\frac{\pi }{2},\frac{\pi }{2}\right]$
Simulation time step (ms)	50
Physics engine time step (ms)	5
Force sensor torque threshold (nm)	1

a See (Faíña et al., 2013) for details.

The best evolved robot for each of the 30 independent runs is selected for transfer. The 30 robots are manually assembled using the visual guiding tool described in section 4.3 and a set of real EMERGE modules, including a base module and several EMERGE modules (AX-18 A version), with their corresponding AprilTags^5^. The assembly time is recorded for each morphology. And the controller is setup to run in the Dynamixel motors, in the same way as in simulation: a sinusoidal generator is assigned to each of the modules according to the genome and its output specifies the motor position goal at each discrete timestep, however, in this case the next position of all modules is sent to the motors directly from the EDHMOR system using a USB to dynamixel bus adapter (USB2AX). All the target positions are sent to the modules in a bulk write operation. The update frequency had to be set to 10 Hz, instead of 20 Hz, as the computer was also performing the tracking of the robot which is time consuming. This caused the servos to vibrate when moving. A smooth operation was achieved by reducing the maximum speed of the motors to 25 rpm. Power and communications are transferred to the base module through a cable and distributed to the modules through their connectors.

A camera setup is then used to pick the base tag from a moving robot and automatically measure the distance it travels. During evaluation, the Apriltag of the base module is replaced by a larger one in order to place the camera higher and increase the size of the arena. Module disconnections are tallied by hand. This process is repeated 5 times for each of the 30 robots selected. After that, data is aggregated, processed and compared to the corresponding robots in simulation. In order to make a fair comparison, the best evolved simulated robots are retested 10 times adding a random Gaussian noise to the position setpoint in their controllers $N\left(\mu =0,\;\sigma^{2}=10^{-4}\right)$. This noise is intended to generate some variability in the simulation results (our simulations are deterministic without it) and simulate the small delays in communication, and thus in movement, that modules can experience during a real test.

### 5.2 Results

**FIGURE 5:**
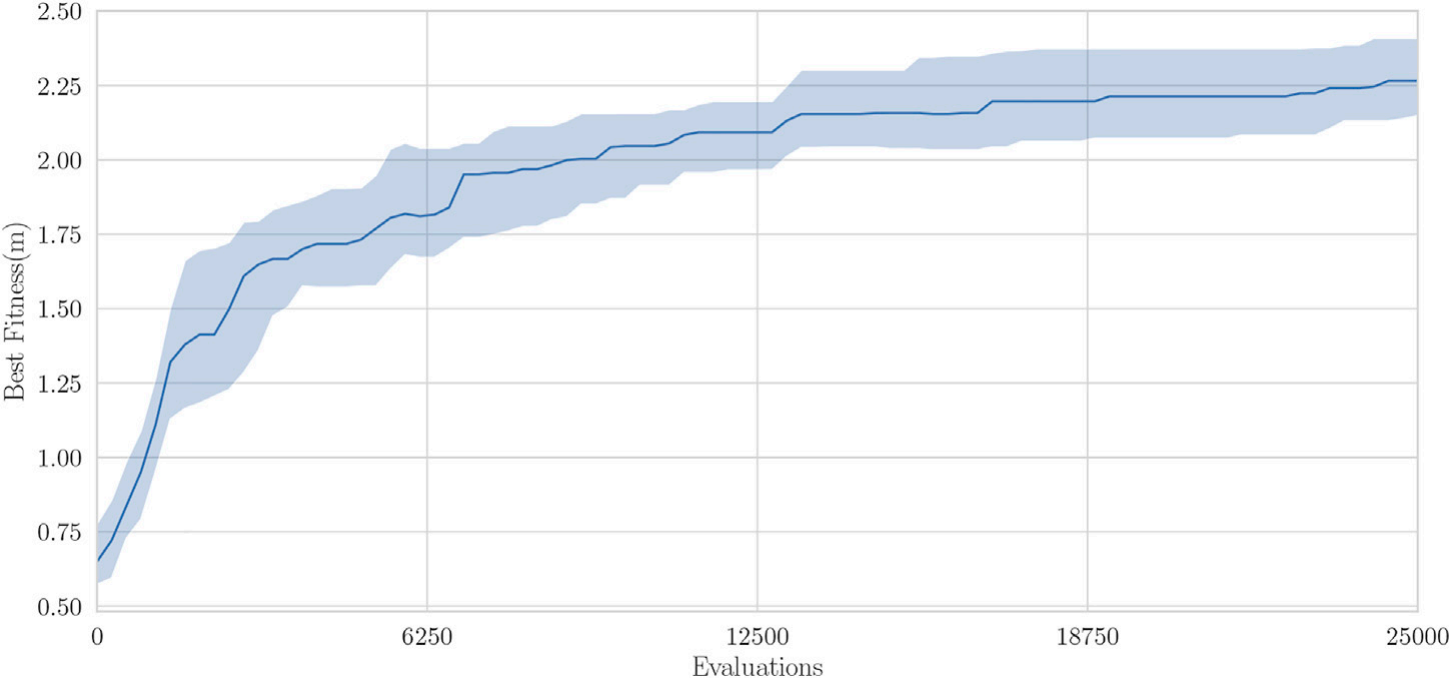
Best individual fitness for 30 runs of EDHMOR in simulation, center line shows the median fitness, the transparent bars show the inter-quartile range.

Figure 5 shows the median best individual fitness of the 30 simulated evolutionary runs against the number of evaluations the algorithm performs. The best individuals have a median fitness of around 2.27 m. Some are even able of traveling over 3 m.

Resulting robots have two or three small limbs attached to the base module, which they use to thrust themselves. Some robot use these limbs in a wheel or rolling like fashion that does not include the base module, while others that have one or more long limbs, use snake like movements that do include the base. At the end of the evolution, the best robots have a median of nine modules including the base, and a median of 0 disconnections.

**FIGURE 6:**
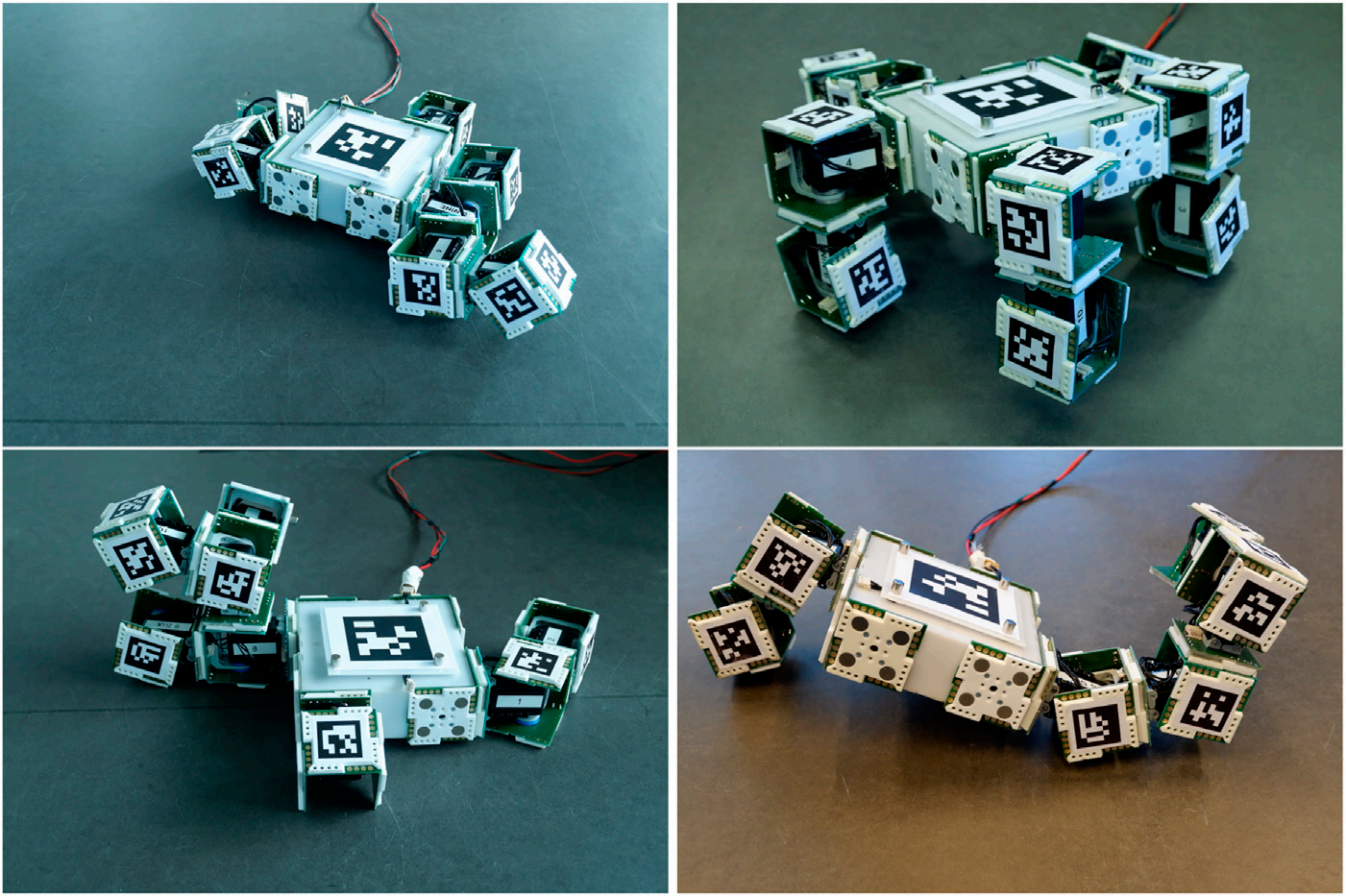
Some evolved robots that were manually built with EMERGE modules with the help of the visual guiding tool. The robots are shown with the motor of the modules set at the initial angle in the evaluation, based on the evolved controllers. All robots took under 5 min to assemble.

**FIGURE 7:**
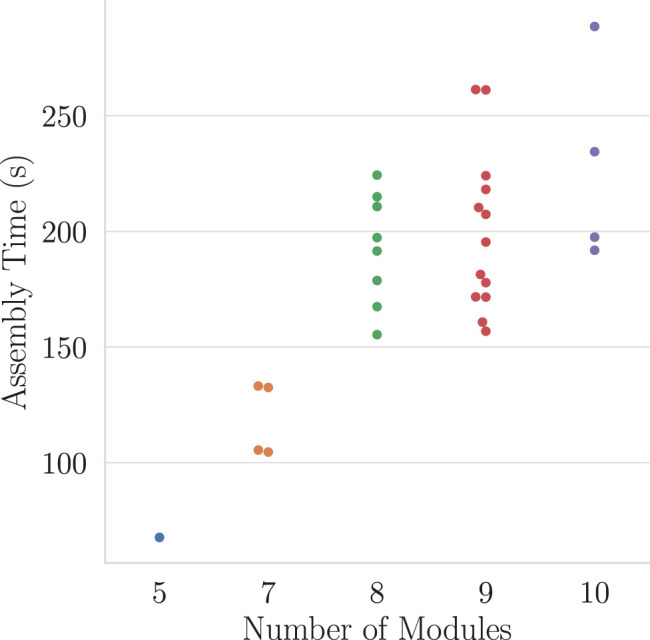
Robot maal assembly time vs. the number of modules. The median time of assembly is 191 s.

All the best simulated morphologies are successfully assembled in reality with the aid of the manual visual tool. Some of the assembled morphologies are shown on Figure 6.^6^ The combination of magnetic connectors and the visual tool makes assembling robots take minutes: All the selected robots take under 5 min to assemble, and the median time is 191 s. Needless to say, the assembly time depends on the number of modules a robot has, and a linear relation can be observed in Figure 7.

**FIGURE 8:**
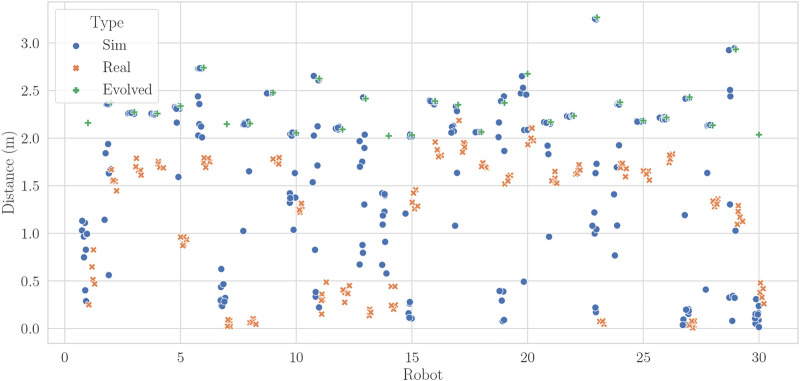
Distance traveled by the robots in reality (Real) and in simulation with noise in the controller (Sim). We have also plotted the original distance traveled by the robot in its evolutionary run (Evolved).

In Figure 8, we have represented the distance traveled of the evolved robots, the real experiments, and the simulation experiments with noise for the best robot obtained in the 30 independent runs. As we can observe, the distance traveled by the robot in the evolution is usually the highest value. There are only a few times where the robot evaluated in simulation with noise travels a bit further that the evolved one. Regarding the experiments performed in reality, the assembled morphologies try to move in similar ways to their simulated counterparts. However, differences in friction between the module materials and ground, the shape of the modules, and small differences in the magnetic forces in the connectors make these movements less efficient and thus the robots are not able to travel as far as the best robots evolved or the ones evaluated in simulation with noise. Low traveled distances in real experiments (less than 0.8 m) are caused by robots where one of their connections detached (robots 1, 8, 11, 23, 27 and 29), robots that did not get enough traction and almost did not move (robots 7 and 14) and robots that moved in a circular pattern and ended up close to where they started (robots 12, 13 and 30). Simulated robots with noise present a high variation in their distance traveled in average (Average interquartile range (IQR) [1.50, 1.98]). This contrasts with the average variation in the distance traveled by the real robots (Average IQR [1.11, 1.17]) which is lower.

**FIGURE 9:**
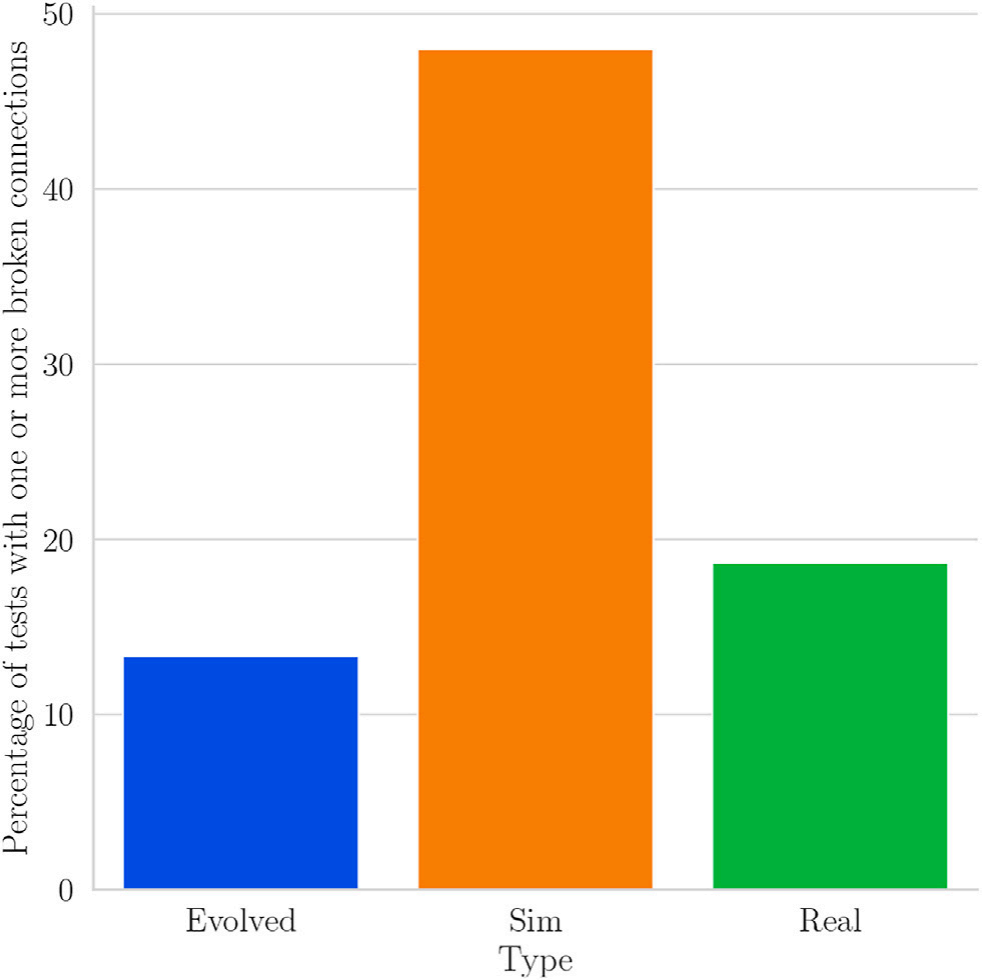
Percentage of tests with one or more broken connections for real robots (Real), simulated robots with noise in the controller (Sim) and the original test performed in simulation in the evolutionary run (Evolved).

The connections between modules are also broken in a higher amount in real robots than in the best robots obtained by evolution. Figure 9 shows the percentage of tests with one or more disconnections in each type of tests performed with the evolved robots. The robots with the highest number of broken connections correspond to the simulated robots with noise, which can be attributed to the random process and to the higher number of tests (300) compared to the other two (Real: 150, Evolved: 30). The high number of broken connections explains the high variability in fitness of robots simulated with noise. In some of the real tests, the disconnections were small enough for the modules to remain electrically connected and for the magnets to quickly snap back the connector to the correct position.

We performed around 300 evaluations of real robots. 150 were performed to collect the data for the five evaluations of the robots. However, we also needed to perform several additional tests for each robot. This was due to the small area available in our tracking system. Thus, two or three tests were used to find a good starting position for a specific robot in order for the path of the robot to be within the limits of the tracking system. Even with this optimization, there were robots that deviated randomly from their path or traveled a distance which was close to the range limit of the tracking system. Thus, several tests needed to be performed to get five valid experiments. This issue needs to be fixed for achieving a full cycle reconfigurable hardware, and this limitation is addressed in the discussion.

**FIGURE 10:**
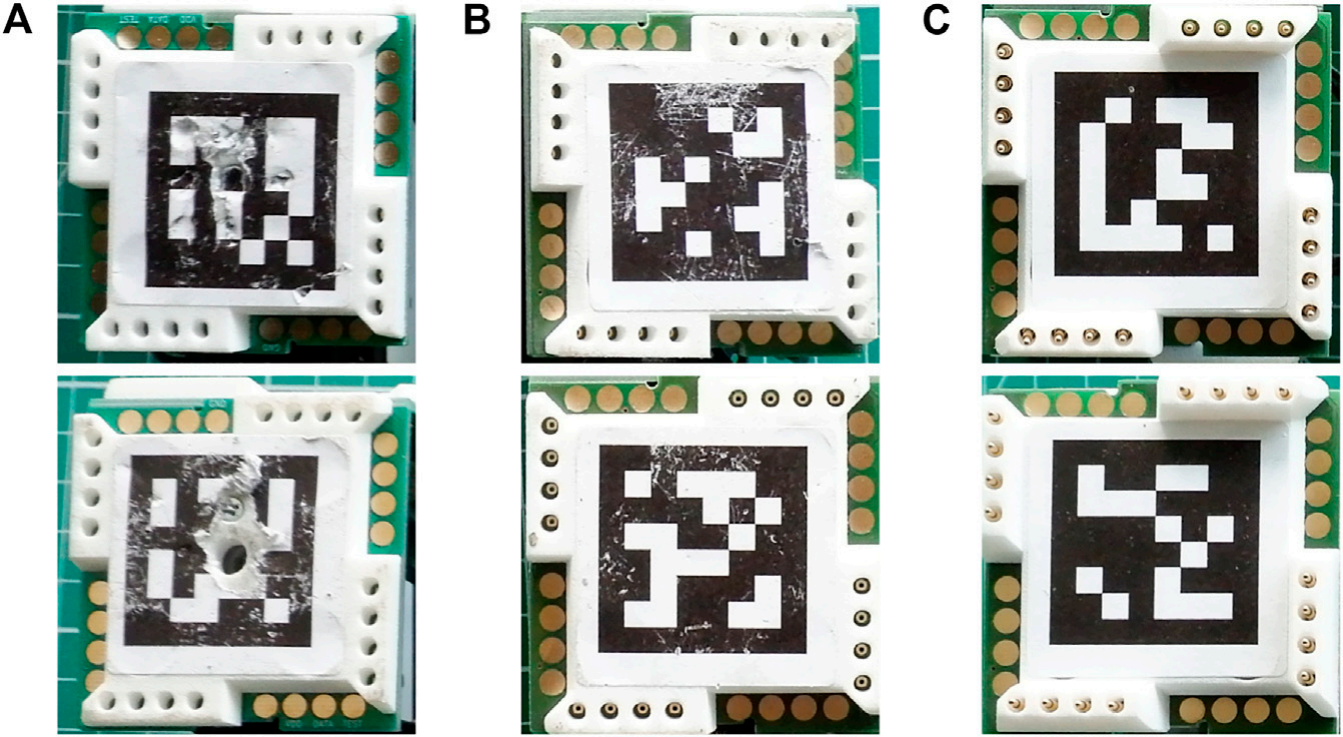
The fiducial markers after approximately 300 physical evaluations **(A)** Three pierced tags on female connectors were found **(B)** Signs of wear on female connectors were found on eight tags **(C)** There was no wear on all male connectors.

In all these 300 evaluations, none of the modules was damaged. Apart from the full disconnections reported in Figure 9, we have observed partial disconnections. In some occasions, the partial disconnection removed the power of the modules, but after being reconnected again the modules continued working without problems. In other cases, the partial disconnection did not remove the power or communications and the module was still moving. The only signs of wear were observed in the fiducial markers (but none of them were replaced during the experiments). Three of them were pierced (see Figure 10). This only happened with specific morphologies where the edge of some modules pushed against the center of the tag which is not supported (there is a hole at the center to be able to access the screw that holds the bushing hub). Surprisingly, these fiducials were still recognized in some orientations. Other eight tags showed wear due to the module slipping on the ground, but they were recognized at all times. Figure 10B shows the most damaged tags that were not pierced. Finally, Figure 10C shows the fiducials on male connectors which did not show any sign of wear or tear.

## 6 Automatic Reconfiguration

The last section showed that robots built using EMERGE modules are able to reproduce to some extent the performance of their simulated counterparts. It has also shown that by standardizing the assembly process, the robot reconfiguration time can be managed down to the order of minutes, even in a manual process, thus opening the possibility of running evolutionary experiments with high numbers of individuals with the same basic building blocks. Standardization is possible thanks to the use within the same system of both the visual guiding tool, which frees the operator from having to check the robot, and the module magnetic connectors.

In addition to protecting from catastrophically failing when faced with high forces, magnetic connectors also allow the quick connection and disconnection of modules from one another. A bending movement with enough torque is all is needed to separate a pair of modules. Since the connectors carry also all the necessary terminals for electrical connections, a recently attached module can start working right away after being placed in a morphology. This quick functional connection and disconnection feature is also the reason a robot can continue working in the face of small disconnections when moving.

EMERGE magnetic connectors have been analyzed regarding assembly and disassembly movements and the forces involved in them (Moreno et al., 2018). Magnetic connectors show a self-aligning force that helps correct misalignments when assembling modules, thus an agent assembling the robot does not have to be highly precise to perform this task. The force acts, although diminishing with distance, at up to 10 mm of misalignment when using ø12 × 3 mm magnets.

The disassembling of the robots can be performed by moving the module that has to be extracted, away from the other modules until the magnetic forces are small enough to move it freely. Again this process can be performed by an imprecise agent, thus making the assembly and disassembly processes easy enough for a non specialized human or even a robotic manipulator to perform. In Moreno et al. (2018), a robot manipulator has demonstrated to perform both automatic assembly and disassembly of EMERGE modules without human intervention. However, the EMERGE modules used were of the previous version, which had ø8 x 3 mm magnets and the connector torque was 0.6 Nm. In addition, the spring pins were at the center of the connector and there was no space for the fiducial markers. As a localization system was required, we attached temporal markers to some connection faces, but these markers obstructed the connector, which was not used in the assembly. Due to limited access to the lab during the pandemic, we were unable to repeat this experiment with the new version of the EMERGE modules and we report the previous results. The new design presented in this article addresses the issue of obstructing fiducial markers by assigning a specific place on the connector for the marker without obstructing the connector.

**FIGURE 11:**
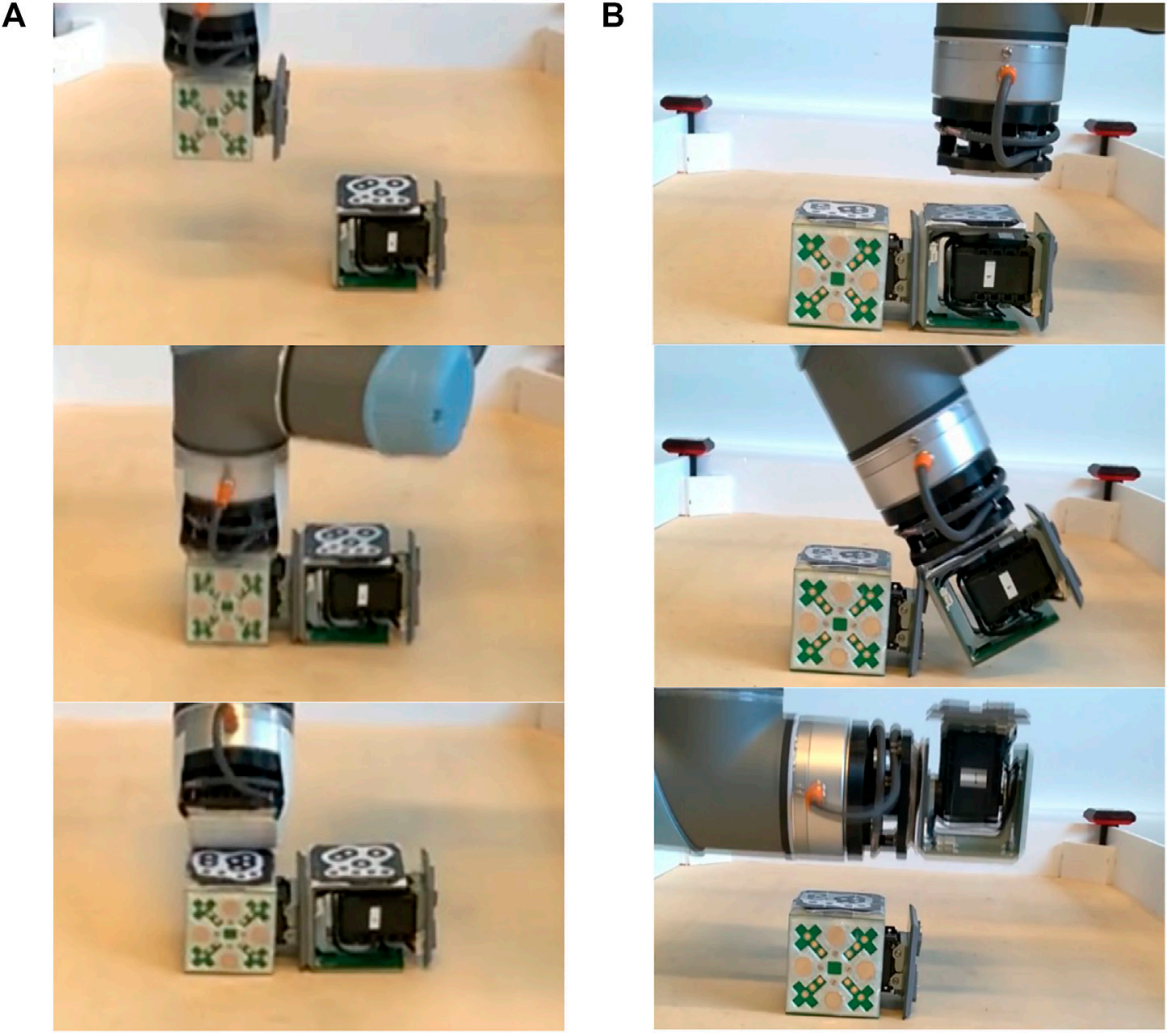
Automatic reconfiguration of the EMERGE modules **(A)** Attachment of a module to another module by using a passive gripper and subsequent release **(B)** Detachment of a module from a morphology using an arc-like movement.

Using ReacTIVision markers (Kaltenbrunner and Bencina, 2007) on selected female connectors, an automatic system tracks the position of the modules inside the manipulator workspace. It is to note that the visual tracking system is a must for performing this operation. To build a morphology from a set of free modules, the manipulator gets close enough to the face of the first selected module to magnetically attach it. The manipulator next transports the module to its designated position, which can be an initial position or a position inside an assembly in progress, the gripper then performs a sequence of movements to detach from the module (see Figure 11) and continues with the next module. This process is repeated with every module until the desired morphology is built.

In the disconnection side, an active tool (i.e. a sharp point) and a passive approach with an arc movement have been successfully used to disassemble robots. The active tool approach requires the sharp edge to be precisely aligned with the middle of the connectors, and it also depends on the specific geometry of the connector faces to slide between without damaging them (see Moreno et al. (2018)), which limits the choice of module geometries that can be used. In contrast, the passive gripper-arc movement approach (see Figure 11B) only requires a leveled surface and the remaining module to be in a position in which it is not moved by the torque exerted. The passive gripper can be built using spare magnets, which again help with alignment, and free the manipulator from having to be completely precise when picking a module. Moreover, the arc-like movement enables the gripper to separate a range of face geometries.

In the passive gripper approach, the manipulator approaches the module of interest in the morphology and attaches to it. The manipulator then performs an arc movement around one of the edges of the connector assembly to be separated. After the connectors have been separated, the manipulator moves the module to a designated position and separates from it in the same way as it did when assembling it to a morphology. The module can also be reassembled into a new morphology instead. Thus, as long as the modules are inside the manipulator workspace, the cycle of reconfiguration can continue occurring indefinitely without external intervention.

## 7 Discussion

Using a robotic manipulator with a passive magnetic gripper, a fully automatic, evolution of morphology in full cycle reconfigurable hardware is on the verge of being achieved. Thus, the EMERGE system opens the door toward full cycle evolution of robots in reality, as has been sought by other systems (Auerbach et al., 2014; Brodbeck et al., 2015; Hale et al., 2019; Nygaard et al., 2019). A representation of the full cycle can be seen graphically on Figure 1. Physical evaluations could be used exclusively, but the system could also be used in conjunction with simulation to run mixed evolutionary processes or techniques to improve the transferability such as (Koos et al., 2012). Furthermore, this system could also be use to implement the Triangle of Life proposed by Eiben et al. (2013).

The new EMERGE modular robot design provides a simple to build module that has been validated to build evolved robot morphologies manually. Davey et al. (2012) have compiled mechanical features about some of the most representative modular robot prototypes, including M-TRAN, ATRON, Polybot, and Superbot. Compared to other modular robot prototypes, EMERGE modules use fewer mechanical parts. EMERGE modules have 31 mechanical components without fasteners (51 counting all the fasteners and 71 counting the electrical parts: spring pins, electrical connectors and cables). Most of the other modular platforms contain over 100 parts, with the exception of Polybot, possessing around 43 mechanical parts.^7^ Most of these components support the high number of actuators for multiple DOF and for special connectors that these systems have, making them also heavy (over 400 g in most cases). A one DOF module like EMERGE can achieve morphologies with several DOF when several modules are provided. Magnetic connectors in the EMERGE module also make docking modules instantaneous compared to modules with self-reconfiguring connectors, even if they are magnetic as with SMORES, and that can take up to 50 s in some cases. SMORES-EP uses electro-permanent magnets that can provide a good alternative over magnets. However, their magnets need to be manually manufactured and aligned precisely (Tosun et al., 2016), which makes them more difficult to manufacture and increases the module building time.

Moreover, when described, other prototypes rarely present assembly instructions, so a time for assembly of each of the modules can not be obtained. However, we estimate that some of them can take hours to complete due to the complexity of their designs, as opposed to the half an hour it takes to assemble an EMERGE module. In our previous EDHMOR system, each module took around a full day of assembly time. Perhaps the most similar design to our work is probably the one made by Krupke et al. (2015). They designed a 3D printed module with off-the-shelf parts and magnetic connections, but it is not designed to support external reconfiguration and its assembly time is high, in their own words: “Surprisingly, the bottleneck in this continuous two-stage verification process is not the printing time itself, but the manual assembly of hardware components.” Some of the mentioned modular robot prototypes have already been used to perform evolutionary experiments, although mostly in the control front (Sproewitz et al., 2008).

Although self-reconfiguring modular robots could be used for morphology evolution, their self-reconfiguration mechanisms would slow the process down or fail to connect in some occasions, adding also complexity to the robot design and building process. Additionally, it is not trivial to design an algorithm to obtain the reconfiguration steps for modular robots (Stoy et al., 2010), specially for three dimensional structures (Liu et al., 2019) and in chain type modular robots (Sucan et al., 2008). Moreover, even though mobile type self-reconfiguring modular robots circumvent some reconfiguration limitations by independently moving modules, external tools or helping modules are necessary to complete some actions (Liu et al., 2019). Furthermore, most other modular robots lack a way of locating their modules in space. Even in the case of taking advantage of self-reconfiguration, this is a very hard problem in itself to solve. Some works have addressed this by using cameras embedded in the modules (Yim et al., 2007b) or by using specialized and expensive motion tracking systems (Liu et al., 2019). On the other hand, manipulating some modular robots with an external manipulator is no straightforward as some of them have exposed parts that can become damaged in the process (Superbot modules) or simply do not have easy to grip surfaces (ATRON modules).

**FIGURE 12:**
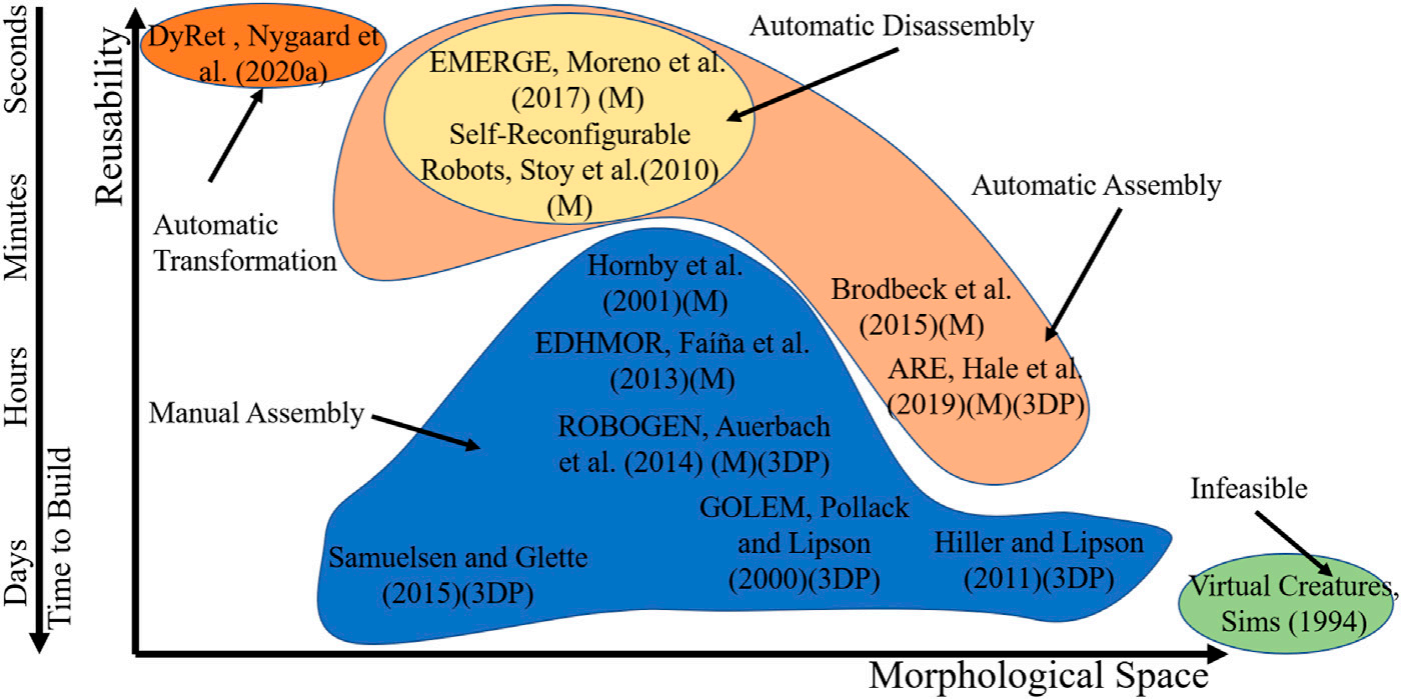
Comparison of real world robot evolution testing approaches. (M) Modular, (3DP) 3D printed.

Among morphology evolution and testing systems, the EMERGE system could provide a way for automatically evolving morphologies from reusable parts. An interesting trade-off exists between the variability of the morphologies that can be created by a system and the reusability of the parts used for building the robot, which also affects the time it takes to build each morphology (Moreno and Faina, 2020a). Figure 12 shows this trade off between morphological variability and deployment time in a graphical way. Modular robots are an interesting middle ground in the morphological space-reusability trade off as all the parts involved in the system are completely reusable if they are not damaged. This contrasts with other approaches like the ARE system described by Hale et al. (2019).

Although EMERGE modules are homogeneous and possess only one DOF, which in the trade-off of Figure 12 limits the morphological variability of the system, this also reduces the time needed for reaching good solutions. Moreover, this also allowed us to standardize and reduce the building time for the module as well as ease the reconfiguration process. Furthermore, complex structures applicable to different problems can still be reached by adding more simple modules to a morphology in different arrangements. Additionally, as the modules are easily modifiable, different types of modules can be designed and added to the system for different applications. We have explored this capability of the system, for example, by varying the length of the modules in simulation in previous works (Liu et al., 2017; Moreno and Faina, 2020b), and expect to test it in reality in future work. For example, we could implement soft parts or passive hinges with springs to make other parts of the robot more resistant to collisions and to store and release energy as human beings do when walking. Additionally, we could also work toward including modules with sensors that allow using closed-loop controllers, as we have explored in (Hernández et al., 2018).

Regarding the assembly disassembly strategy for evolution of morphology in full cycle reconfigurable hardware, other systems can build robots but not disassemble them automatically, e.g. Brodbeck et al. (2015). These two features will allow the EMERGE system to be more autonomous as it does not require a constant input of parts from an external source. It also reduces the amount of waste generated. One limitation is that the system is not as morphologically variable as other systems that use 3D printing and other approaches. Nevertheless, it can assemble limbed and other configurations that show interesting movements and which are comparable to systems that allow more free shapes (Auerbach et al., 2014; Hale et al., 2019; Brodbeck et al., 2015). Morphologies achieved with EMERGE are still more variable than those achieved by shape changing robots (Nygaard et al., 2019), which can be considered to be on the other end of the morphological space-reusability trade off. Shape changing robots allow more powerful actuators to be mounted on robots and are capable even of changing their shape online, also enabling morphology evolution in the real world, however they are also restricted to smaller variations of morphology.

One feature of almost all the systems mentioned, and also of the EMERGE platform, is that the robots built move by thrusting their parts against the environment. This causes forces that can damage robot parts. 3D printed chassis or links can break apart when colliding with walls or the ground itself. Shape changing robots (Nygaard et al., 2019) and some systems that have wheels (Auerbach et al., 2014; Hale et al., 2019) can minimize this damage, but will eventually face a torque or force high enough to break the parts apart. Remaking 3D printed parts for a robot morphology can take as much time as building the robot from scratch. EMERGE modules can be more easily repaired in case of damage by just swapping off the shelf components or 3D printed parts general for all modules and, furthermore, magnetic connectors help them fail in a non-catastrophic way in case of facing high forces. In most cases, instead of repairing a robot from scratch, modules are just quickly reassembled.

Conversely, magnetic connectors are weaker than other types of joints and can lead to a higher number of disconnections than when using stronger or more rigid connectors, such as the ones used in other systems to join the robot parts, which in turn limits the performance of the robots. As an example, evolving EMERGE robots with a higher torque threshold in the force sensors (1.7 Nm instead of 1 Nm and disconnection if 10 consecutive violations are found instead of 7) led to the best robots reaching close to 4 m of distance (median fitness: 3.78 m, fitness IQR [3.48, 4.42]m, median number of modules: 10, median number of broken connections: 0). Robots also showed more violent movements, like rolling the base module, that can better take advantage of the full torque of the Dynamixel motors. A connector that mixes the quick connection/disconnection and robustness capabilities of magnetic connectors with a higher tolerance for external torque is thus highly desirable.

After approximately 300 physical evaluations, the only sign of wear was found on the fiducial markers. However, they can be easily replaced. In any case, we should protect the tags to avoid having to change them very often. Protection against friction with the floor can be easily implemented by adding some protrusions on the female connector (four small parts attached the 16 holes on the female connector). Protection against piercing could be implemented by filling the gaps under the tags.

While we are on the verge of completely deploying a system that implements an evolution of morphology in full cycle reconfigurable hardware, some challenges remain. The first one is the planning of manipulator movements to transport, assemble and disassemble modules in arbitrary 3D morphologies automatically, a problem that, although very studied in industrial and automation settings, must be adapted to the modular robot case. Another challenge is the limited size of the arena in which real robots are tested. Some of the real robots transferred were close to the limits of the space that the camera was able to track (around 2 × 1 m) and the workspace for small manipulators is even less (0.5 × 1 m with an UR5). Other works have solved this problem by making the robot automatically return in case of reaching the limits of the space (Samuelsen and Glette, 2015), pulling cables tied to the robot or resetting the position of the robots manually. However, none of these systems can easily be applied to automatic systems in which the morphology changes. Catching the runaway robot with the manipulator, an annular arena around the manipulator or implementing a dynamic arena that moves under the robot while it is being tested can come in hand for solving this problem. Reinforcing the fiducial markers and using strategies to detect them in case of being obscured by other modules is also a must for increasing the reliability of the system. Finally, as evolutionary runs take thousands of evaluations to complete, eventually some modules will become damaged, a way of detecting the damaged modules and setting them apart for repair would help complete these runs without interruption, making the system more robust.

## 8 Conclusion

This work presented a platform for evolution of morphology in full cycle reconfigurable hardware: The EMERGE modular robot platform. EMERGE is based on robotic modules and eases the reconfiguration process enough so that an external manipulator could run a full cycle process of reconfiguration and testing, i.e. assemble modules in morphologies, test the morphologies, disassemble modules and start all over again. Three necessary parts to implement this approach were described: the mechanical design of the EMERGE module, extensive tests of the modules by first assembling them manually, and automatic assembly and disassembly tests.

As part of the evolution of morphology in full cycle reconfigurable hardware perspective, the newly designed EMERGE modules are fast to build, and we have shown that one module can be built in half an hour. Modules are constructed from off-the-shelf and 3D printed parts, and a soldering iron and a few screwdrivers are the only necessary tools. This enables the creation of multiple modules in a reasonable amount of time and makes them accessible to a bigger audience.

Thanks to using magnetic connectors, the modules are quickly attached and detached to assemble robotic morphologies and to reconfigure them manually. A self-aligning force frees the assembling agent from having to be highly precise. Manual assembly times were found to take under 5 min for robots with less than 10 modules and increase linearly with the number of modules in the morphology. Thus, the system would be able to support morphologies with a high number of modules with a reasonable impact on assembly time.

Manual assembly for testing the module design is aided by a visual guiding tool that checks the modules positions in the morphology by comparing each subsequent module with their simulated counterparts and providing feedback on whether everything is correct. This tool eases the assembly of robots by an operator, freeing the need to check the correct positioning of module and thus reducing the time for assembly and reconfiguration.

Extensive tests of evolved morphologies transferred manually to reality showed that, taking the reality gap into account, real EMERGE modules are able to reproduce to some extent the performance of their simulated counterparts, both in the distance traveled and in the number of disconnections in each test. Tests also showed that magnetic connectors make robots more resilient against catastrophic failures as modules just disconnect in case of being exerted by high external torques and forces and only need to be reassembled to continue working. Although sustaining some damage, fiducial markers also help automatically track the position of the robot in the test and are easy to replace.

Module tracking combined with the easy assembly and disassembly provided by the use of magnetic connectors enables EMERGE modules to be also reconfigured using an external robotic manipulator. The new design makes tracking an integral part of the module. Experiments have demonstrated that it is possible to attach and detach modules from a morphology, as well release the module from the manipulator with a passive magnetic gripper. Automatically reconfiguring an EMERGE morphology with a manipulator opens up the possibility of running completely autonomous, full cycle (assembly testing-disassembly), evolution of morphology and control in robots physically or with mixed simulation-reality approaches.

Future work includes devising a general planer of manipulator movements for assembling and disassembling arbitrary 3D robot morphologies, to increase the autonomy of the system for doing evolutionary experiments. Additionally, creating an annular arena or an automatic arena for allowing modules to move freely in constrained spaces can help run more complete tests. Finally, a way of tracking module health and setting modules aside for maintenance can also increase the system robustness.

## Data Availability

The raw data supporting the conclusion of this article will be made available by the authors, without undue reservation.

## Footnotes

1 The open source designs and fabrication files of the modules can be found at https://sites.google.com/view/emergemodular/home.

2 Genderless connectors could be designed by using male connectors in all the faces and placing eight magnets with different polarities instead of four with the same polarity.

3 A video of the assembly process can be found at https://vimeo.com/afaina/emerge-assembly.

4 CoppeliaSim can be downloaded free for educational purposes from https://www.coppeliarobotics.com/.

5 The source code of the simulation experiments, as well as the visual guiding tool and the real robot controllers, can be found in https://bitbucket.org/afaina/edhmor/src/master/.

6 Videos of the robots can be found at https://vimeo.com/afaina/emerge-evolved-robots.

7 We are not sure if they include fasteners in this calculation as the original source is not available.
